# Disseminated Cutaneous Herpes Zoster in an Immunocompetent Patient: A Diagnostic Challenge

**DOI:** 10.7759/cureus.102786

**Published:** 2026-02-01

**Authors:** Meriah Frauwirth, Randall Scott

**Affiliations:** 1 Dermatology, Lincoln Memorial University-DeBusk College of Osteopathic Medicine, Knoxville, USA; 2 Family Medicine, Family Wellness Center, Green Cove Springs, USA

**Keywords:** diagnostic challenge, disseminated herpes zoster, immunocompetent adult, varicella-zoster virus, vesicular rash

## Abstract

Disseminated cutaneous herpes zoster (DCHZ) typically occurs in immunocompromised individuals. However, rare cases arise in immunocompetent adults. These cases may initially resemble benign dermatoses or be difficult to distinguish from other systemic rashes when overlapping risk factors obscure diagnosis. Early recognition is essential to prevent visceral or neurologic complications.

A 32-year-old immunocompetent male with childhood varicella presented with a 10-day history of a pruritic vesicular rash, right ear fullness, and viral prodromal symptoms. He had recent diving exposure five days prior. The eruption began with axillary myalgia progressing to vesicles spanning multiple right thoracic dermatomes, eventually extending anteriorly. Folliculitis was excluded due to non-pustular morphology and preceding neuropathic discomfort. Decompression-related skin injury was ruled out, given symptom onset and absence of cutis marmorata. Physical examination demonstrated grouped vesicles beyond contiguous dermatomes, consistent with DCHZ. The patient was treated with valacyclovir and gabapentin.

DCHZ in immunocompetent adults is uncommon but may lead to systemic complications. Misdiagnosis is possible when early lesions mimic folliculitis or irritant dermatoses. If relevant risk factors are present, such as recent deep or prolonged diving exposure or inadequate decompression, the presentation may have other cutaneous changes, making clinical differentiation challenging. Key distinctions in lesion morphology, distribution, and associated neuropathic symptoms are essential for separating these mimickers and recognizing disseminated varicella-zoster virus (VZV) promptly.

This case highlights that disseminated cutaneous herpes zoster may occur in healthy adults and underscores the importance of early diagnosis and prompt antiviral therapy, particularly when risk factors are present, and lesion morphology mimics benign conditions.

## Introduction

Herpes zoster (HZ) is one of the most well-known dermatologic manifestations that is classically described with a dermatomal rash. Herpes zoster is understood to be caused by the reactivation of latent varicella-zoster virus (VZV) within sensory ganglia. This leads to a pathognomonic unilateral vesicular rash confined to a single dermatome, without crossing the body’s midline. Disseminated cutaneous herpes zoster (DCHZ) is defined as having ≥20 vesicular lesions outside the primary or adjacent dermatomes and traditionally occurs in immunocompromised hosts [1,2]. However, rare cases in immunocompetent adults exist and establish that viral dissemination can occur even without immune dysfunction [2-4]. 

Disseminated herpes zoster can lead to life-threatening complications with potential visceral organ involvement and neurologic complications; therefore, early recognition and antiviral treatment are critical [5,6]. Clinical management and diagnosis may be challenging due to unusual clinical presentation, symptom onset, or relevant risk factors for other systemic rashes. This case highlights a rare case of disseminated herpes zoster in an immunocompetent adult and emphasizes the diagnostic challenges and importance of timely antiviral therapy. 

## Case presentation

A 32-year-old male, with a childhood history of varicella infection at age four, presented with a 10-day history of a pruritic, erythematous vesicular rash and right ear fullness. The patient was a frequent diver with an involved underwater activity and had last descended five days prior to his office visit. He reported right axillary muscle pain that was unlike the typical soreness he experiences after diving sessions. He also admitted to headaches and sinus congestion, suggesting a possible viral upper respiratory prodrome. He denied dizziness, confusion, nausea, or vomiting.

**Figure 1:**
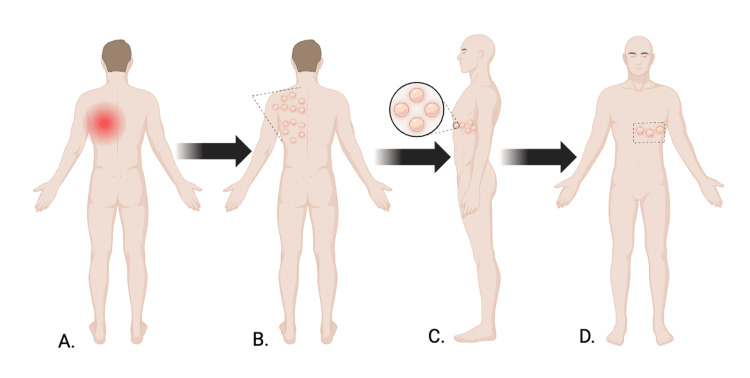
Progression and distribution of vesicular eruptions Schematic illustration demonstrating the temporal progression and anatomic distribution of the patient’s cutaneous findings. Panel A depicts localized neuropathic discomfort preceding rash onset. Panel B shows clustered vesicular lesions involving multiple right posterior thoracic dermatomes. Panel C provides a magnified view of grouped vesicles. Panel D illustrates subsequent anterior extension below the nipple line. Arrows indicate areas of interest, including posterior involvement, anterior progression, and satellite lesions beyond contiguous regions, consistent with disseminated cutaneous herpes zoster. Created in BioRender. Frauwirth, M. (2026) https://BioRender.com/joz7k3o

The eruption began with localized axillary myalgia, followed by the appearance of itchy, mildly painful vesicles over the right posterior thoracic region. Over several days, the lesions crusted and partially healed, while new vesicles appeared and progressively spread across multiple right thoracic dermatomes. The rash subsequently extended anteriorly, following the dermatomal distribution to the chest below the nipple line (Figure 1). The eruption consisted of more than 20 vesicular lesions extending beyond the primary and adjacent dermatomes, meeting criteria for disseminated cutaneous herpes zoster. He denied fever, burning, tingling, or other systemic symptoms. Symptomatic measures such as epsom salt baths and ibuprofen provided minimal relief. 

**Figure 2:**
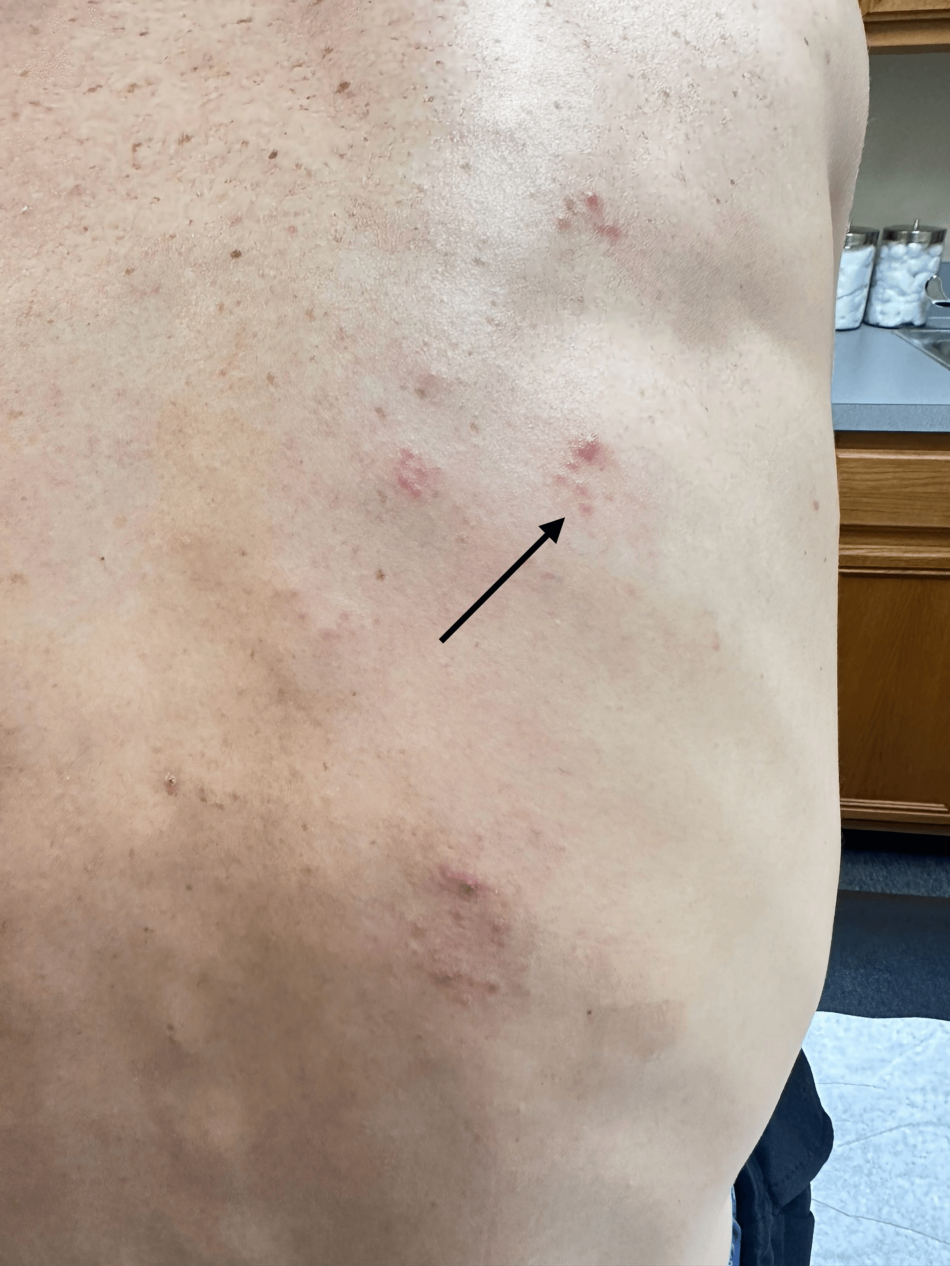
Disseminated clusters of vesicles involving multiple thoracic dermatomes Clinical photograph demonstrating clustered vesicular lesions distributed across multiple right thoracic dermatomes, consistent with disseminated cutaneous herpes zoster. The image demonstrates grouped vesicles accompanied by satellite lesions extending beyond adjacent dermatomal boundaries.

A comprehensive clinical evaluation was initiated to determine the underlying etiology. Folliculitis was excluded as the lesions were vesicular rather than pustular, were not centered around hair follicles, and the presence of localized neuropathic pain preceding the rash was inconsistent with a bacterial process (Figure 2). The more serious diagnosis of decompression-related skin injury (“the bends”) was also ruled out, as the rash lacked the mottled or marbled pattern typically associated with nitrogen embolization. Further, the lack of neurologic deficits and the timeline for serious illness, even considering delayed diagnosis, was well past 24-48 hours of typical occurrence (Table 1). 

**Table 1:** Clinical comparison of folliculitis, decompression injury, and disseminated cutaneous herpes zoster Differential diagnosis chart comparing folliculitis, decompression-related injury, and disseminated cutaneous herpes zoster (DCHZ). The figure outlines each condition’s definition, risk factors, clinical presentation, and prognosis, highlighting distinguishing features used in clinical evaluation of vesicular or mottled eruptions [2,7,8].

Disease	Folliculitis	“The Bends” (Decompression Sickness)	Disseminated Cutaneous Herpes Zoster (DCHZ)
Cause	Superficial inflammation or infection of hair follicles, commonly caused by Staphylococcus aureus	Nitrogen gas embolization due to rapid decompression after diving	Reactivation of latent varicella-zoster virus- widespread vesicular eruption involving ≥20 lesions beyond the primary or adjacent dermatomes
Risk Factors	Friction, occlusion, moisture, or bacterial contamination after shaving or water exposure	Deep or prolonged dives, inadequate decompression, repetitive diving	Typically immunocompromised states, though, may occur in transient immune dysregulation or viral prodrome in immunocompetent adults
Presentation	Small, erythematous pustules centered around hair follicles, often pruritic or tender	Mottled or marbled reddish-purple rash (cutis marmorata) with joint pain, dizziness, or neurologic symptoms	Grouped vesicular lesions on an erythematous base following multiple dermatomes, usually confined to one body side
Prognosis	Generally self-limited	Life-threatening	Potentially serious
Management	Warm compress, avoid irritants, topical antibiotics	Hyperbaric oxygen therapy needed	Early antiviral therapy prevents visceral or neurologic complications

Physical examination revealed clustered vesicular lesions involving multiple thoracic dermatomes with several satellite vesicles beyond contiguous regions, consistent with disseminated cutaneous herpes zoster (Figure 2). Furthermore, the distribution confined to the right thoracic dermatomes, accompanied by localized muscle discomfort and progression along contiguous dermatomes, was most consistent with herpes zoster infection. Taken together, the clinical presentation strongly suggested a diagnosis of disseminated herpes zoster infection. The patient’s prior varicella infection, along with the characteristics of the skin lesions and associated symptoms, reinforced the diagnosis of disseminated cutaneous herpes zoster. The patient was started on valacyclovir 1 gram orally three times daily for 14 days and gabapentin 300 mg daily for neuropathic discomfort, with subsequent improvement in pain and gradual resolution of the vesicular lesions.

## Discussion

Disseminated herpes zoster in immunocompetent adults is rare but potentially life-threatening if not promptly recognized and treated, and is generally defined as the presence of 20 or more vesicular lesions occurring outside the primary or adjacent dermatomes [2,3]. Systemic dissemination can lead to serious neurologic complications, including but not limited to the notorious Ramsay Hunt syndrome, Bell’s palsy, or encephalitis, as well as systemic complications such as hepatitis or pneumonitis in otherwise healthy individuals [5,6]. The early presentation of skin manifestations may complicate the diagnosis. This is because early presentation may mimic benign dermatoses such as folliculitis or contact dermatitis. In cases with prevalent risk factors, such as this patient, decompression-related skin changes may be considered as differential. The simultaneous involvement of multiple dermatomes is not the most common presentation and may lead to overlooking herpes zoster infection and delaying diagnosis. Delayed diagnosis increases the potential for ongoing viral replication, thereby increasing the risk of systemic involvement. Therefore, when evaluating unilateral or disseminated dermatomal eruptions with vesicles extending beyond a single dermatome or associated neuropathic pain, it is important to consider herpes zoster infection. Early initiation of high-dose antiviral therapy is the mainstay of management, limiting viral replication and preventing potential visceral complications [1,5].

Reports suggest that aging is the primary risk factor for disseminated varicella-zoster virus infection in immunocompetent hosts [3,9]. However, in our case, disseminated VZV occurred in a younger male. An alternative explanation for disseminated cutaneous herpes zoster in younger immunocompetent individuals may be related to transient changes in immunity, specifically viral infections or physiological stress. These factors may cause brief suppression of VZV-specific T-cell immunity, allowing viral reactivation and dissemination in otherwise healthy hosts, such as our patient [3,10]. This patient’s upper respiratory viral prodrome may have contributed to the reactivation, or the stress-related diving may have acted as a catalyst for this event. However, it would be challenging to specify whether one event had more influence than the other. While DCHZV in immunocompetent adults is rare, a greater understanding of the mechanisms and risk factors that may contribute to the disease process is critical. This case illustrates how disseminated herpes zoster in an immunocompetent adult can be clinically difficult to distinguish from more common cutaneous conditions, reinforcing the importance of thoughtful diagnostic evaluation, early recognition, and prompt antiviral intervention, while also raising consideration of preventive strategies such as vaccination.

## Conclusions

This case demonstrates the diagnostic challenge posed by disseminated herpes zoster in an immunocompetent adult, especially when the eruption resembles benign skin disorders or when coexisting factors predispose the patient to alternative rash etiologies. Identification requires thoughtful evaluation of lesion morphology, distribution, and symptom progression. Clinicians should consider DCHZ when vesicles extend beyond a single dermatome, particularly in the setting of viral prodrome or life stress. Clinicians should recognize that dissemination can occur even in healthy adults, and timely antiviral therapy is essential to prevent systemic spread and associated complications. 
